# A Novel Application of Serum Creatinine and Cystatin C to Predict Sarcopenia in Advanced CKD

**DOI:** 10.3389/fnut.2022.828880

**Published:** 2022-02-25

**Authors:** Yu-Li Lin, Chih-Hsien Wang, I-Chen Chang, Bang-Gee Hsu

**Affiliations:** ^1^Division of Nephrology, Hualien Tzu Chi Hospital, Buddhist Tzu Chi Medical Foundation, Hualien, Taiwan; ^2^School of Medicine, Tzu Chi University, Hualien, Taiwan

**Keywords:** creatinine, cystatin C, sarcopenia, chronic kidney disease, skeletal muscle

## Abstract

Sarcopenia is highly prevalent in patients with advanced chronic kidney disease (CKD), yet a reliable serum index has not been established. The product of serum creatinine and the estimated glomerular filtration rate based on cystatin C (Cr×eGFRcys) was recently proposed as a sarcopenia index (SI), approximately to 24-h filtered creatinine through the glomerulus. We aimed to evaluate the diagnostic validity of the novel SI in advanced CKD. In 297 patients with non-dialysis stage 3b-5 CKD, aged 68.8 ± 12.9 years, the total skeletal muscle mass (SMM), handgrip strength (HGS), and usual gait speed were assessed. Sarcopenia was defined based on the Asian Working Group for Sarcopenia 2019 consensus update. The prevalence of sarcopenia in this cohort was 20.2%. The SI correlated moderately with SMM (*r* = 0.503, *P* < 0.001), HGS (*r* = 0.508, *P* < 0.001), and gait speed (*r* = 0.381, *P* < 0.001); the independency of the SI with three muscle metrics was confirmed after extensive adjustment. For sarcopenia prediction, the SI had acceptable discriminative powers in males [area under the receiver operating characteristic curve (AUC) 0.646, 95% confidence interval (CI) 0.569–0.718] and females (AUC 0.754, 95% CI 0.670–0.826). In males, the best cut-off was 53.9, which provided 71.1% sensitivity, 58.0% specificity, 32.9% positive predictive value (PPV), and 87.4% negative predictive value (NPV); in females, the best cut-off was 45.8, which provided 81.8% sensitivity, 62.3% specificity, 31.0% PPV, and 94.3% NPV. In conclusion, Cr×eGFRcys could be served as a surrogate marker for sarcopenia and may be helpful for sarcopenia screening in advanced CKD. Further studies are needed to expand our investigation.

## Introduction

Sarcopenia, which is characterized by a progressive decline of skeletal muscle mass, strength, and physical performance, is frequently observed in patients with chronic kidney disease (CKD) and leads to poor clinical outcomes (1–3), as pathogenic factors—metabolic acidosis, inflammation, impaired insulin signaling, oxidative stress, accumulated uremic toxins, suppressed appetite, decline in satellite cells, and myostatin overexpression—accelerate skeletal muscle wasting as kidney disease progresses (4, 5). Thus, sarcopenia is a major concern in patients with advanced-stage CKD. Moreover, protein restriction is usually implemented in patients with advanced-stage CKD to attenuate renal progression, and clinical supervision of nutritional status is highly recommended (6). Imaging modalities including bioelectrical impedance, ultrasound, dual-energy X-ray absorptiometry, computed tomography, and magnetic resonance imaging, as well as D_3_-creatine dilution, are strongly recommended by expert panels as measurement tools for skeletal muscle mass (7–10), yet are not always available in outpatient settings. Therefore, surrogate markers for the early detection of sarcopenia in advanced-stage CKD are in demand.

Creatinine is a metabolite of creatine phosphate that is converted non-enzymatically from skeletal muscle, and the endogenous creatinine generation rate depends on skeletal muscle mass under steady renal function (11, 12). Due to its properties of free glomerular filtration and minimal tubular reabsorption, timed urine creatinine excretion (Ucr) is a reliable marker for predicting skeletal muscle mass in various populations (13–16). Unfortunately, the collection of 24-h urine samples is inconvenient. Moreover, under- or over-collection of urine samples is common.

To overcome the need of precise urine collection, 24-h filtered creatinine through the glomerulus can be estimated from the product of serum creatinine (Cr) and the glomerular filtration rate (GFR). Through the calculation of GFR from cystatin C (eGFRcys), a renal marker less affected by skeletal muscle mass than creatinine, Cr×eGFRcys has been proposed as a novel sarcopenia index (SI) (17). Our previous work demonstrates a significant impact of the low SI on overall mortality in non-dialysis CKD patients (18). However, whether this index is useful for the evaluation of skeletal muscle mass and strength in patients with advanced-stage CKD remains unexplored.

Thus, the study aimed to investigate the association of the novel SI with skeletal muscle mass, strength, and physical performance in patients with stage 3b-5 CKD and to establish its diagnostic validation for sarcopenia.

## Materials and Methods

### Participants

This cross-sectional study was conducted in outpatient clinics at Hualien Tzu-Chi Hospital, a Medical Center in eastern Taiwan, between January 2018 and May 2021. Adult patients with non-dialysis stage 3b-5 CKD who have regularly been followed up at our CKD outpatient department were invited to participate in the study. Those younger than 20 years, with an estimated GFR of more than 45 mL/min/1.73 m^2^, recent hospitalization within 3 months, a pacemaker, amputated limbs, active malignancy, wheelchair or bedridden, as well as those who refused to participate were excluded. CKD was defined as a decrease in renal function or the presence of kidney damage for more than 3 months (19).

A total of 297 patients with CKD were enrolled in this study. Basic information and comorbidities, including diabetes mellitus (DM), hypertension, chronic glomerulonephritis (GN), and cardiovascular (CV) disease, were collected through electronic medical records. CV disease comprised coronary artery disease, myocardial infarction, left ventricular hypertrophy, arrhythmias, or congestive heart failure.

All participants signed an informed consent approved by the Institutional Review Board of Tzu-Chi Hospital (IRB 108-219-A), and all methods were performed in accordance with the Declaration of Helsinki.

### Anthropometric Analysis and Blood Pressure

Body mass index (BMI) was calculated as body weight (Kg) divided by height squared (m^2^). In the standing erect position, waist circumference was measured at the shortest point between the lower rib margin and the iliac crest; hip circumference was measured at the level of greatest protrusion of the buttocks. Triceps skinfold (TSF) and mid-arm circumference (MAC) were measured at the midpoint between the acromion and olecranon using a skinfold caliper (QuickMedical, Issaquah, WA, USA) and flexible inextensible tape, respectively. The mean of the three TSF readings was accepted. Mid-arm muscular circumference (MAMC) was subsequently calculated as MAC (cm)-π × TSF (cm).

Systolic and diastolic blood pressure (BP) were measured using standard mercury sphygmomanometers after 10-min resting.

### Skeletal Muscle Mass, Handgrip Strength, and Gait Speed

Skeletal muscle and fat tissue mass were assessed using a tetrapolar bioelectrical impedance device (Biodynamics® BIA 450 Bioimpedance Analyzer, Seattle, WA, USA), which delivers an electric current of 800 μA at 50 kHz. In the supine position, two electrodes were placed on the hand and wrist, and two were placed on the foot and ankle of the non-dominant side. Total skeletal muscle mass (SMM) was estimated based on a well-validated equation developed by Janssen et al. (20):


$$\begin{array}{l} \text{SMM}=\left(\frac{{\text{height}}^{2}}{\text{resistance}}\times 0.401\right)+\text{age}\times \left(-0.071\right)+\text{sex}\\ \;\times \;3.825+5.102 \end{array}$$

In this formula, height is input in centimeters; resistance in ohms; age in years; sex: female = 0, male = 1.

A hand-held dynameter (Jamar Plus Digital Hand Dynamometer, SI Instruments Pty Ltd, Hilton, Australia) was used to assess the handgrip strength (HGS). Patients were instructed to grip the dynamometer as tightly as possible in the standing position, with the arm at a right angle and the elbow at the side of the body. Three measurements were repeated in each hand, with a 1-min rest interval. The average value of both hands was adopted for analysis.

For the usual gait speed measurement, participants were instructed to walk at their usual speed for 6 m on a flat and straight path, and the gait speed was calculated accordingly. The gait speed test was not performed on 22 patients who reported dizziness or an unsteady gait on the test day.

### Definition of Sarcopenia

The skeletal muscle index (SMI) was calculated as the SMM (kg) divided by height squared (m^2^). Those with an SMI lower than 8.87 kg/m^2^ in males and 6.42 kg/m^2^ in females were classified as having a low SMI, based on two standard deviations below the mean of young Taiwanese adults (21, 22). Muscle weakness was defined as an HGS < 28 kg in males and 18 kg in females, whereas slow gait speed was 6-m gait speed < 1.0 m/s, based on the Asian Working Group for Sarcopenia (AWGS) 2019 consensus (23). Sarcopenia was defined as low SMI with either muscle weakness or slow gait speed.

Among 22 patients who did not perform the gait speed test, none of them had low SMI. Thus, all of them were classified as non-sarcopenic.

### Serum SI and Laboratory Data

At the same visit, fasting serum samples were used for biochemical analysis within 1-h of collection. A standard autoanalyzer (Siemens Advia 1800, Siemens Healthcare GmbH, Henkestr, Germany) was used to determine serum blood urea nitrogen (BUN), creatinine, albumin (bromocresol green method), total cholesterol (TCH), glucose, and urine protein/creatinine ratio (UPCR). Serum cystatin C levels were measured using a nephelometric Siemens immunoassay. The estimated GFR was calculated from serum creatinine (eGFRcre) and cystatin C (eGFRcys), based on the Modification of Diet in Renal Disease (24) and CKD-EPI Cystatin C equation (25), respectively. The stages of CKD in the study were based on the eGFRcys.

The novel SI, Cr×eGFRcys, was calculated as the product of serum creatinine (mg/dL) and eGFRcys (mL/min/1.73 m^2^).

### 24-H Urine Creatinine Excretion

Detailed verbal and written instructions about the urine collection technique were provided to all participants. After discarding the first void in the morning, all participants were instructed to collect all urine throughout the following 24-h period, including the first morning void on the next day. The 24-h Ucr was calculated as the product of urine creatinine levels and 24-h urine volume. Among our participants, 265 (89.2%) completed 24-h urine sample collection.

### Statistical Analyses

To detect a correlation coefficient of about 0.3 between SI and skeletal muscle measures in each gender, with an alpha level of 0.05 and a power of 90%, a total of at least 224 patients should be enrolled.

Continuous variables were expressed either as the mean ± standard deviation or as the median and interquartile range, based on the data distribution evaluated from the Kolmogorov–Smirnov test. The variables among sarcopenia and non-sarcopenia were compared by applying Student's independent *t* test or the Mann–Whitney U test. Categorical variables were expressed as absolute (*n*) and relative frequency (%) and were analyzed by the chi-square test. Scatter plots with Spearman's correlation coefficient were used to depict the correlations of SI and 24-h Ucr with SMM, HGS, and gait speed. Independency of SI with SMM, HGS, and gait speed was examined by multiple linear regression, adopting potential risk factors for sarcopenia.

To assess the diagnostic performance of SI and 24-h Ucr on sarcopenia, receiver operating characteristic (ROC) curves were constructed. The area under the ROC curve (AUC), cut-offs, sensitivity, specificity, positive predictive value (PPV), and negative predictive value (NPV) were established.

Statistical analyses were performed using SPSS (version 19.0; SPSS, Chicago, IL, USA). A *P*-value of < 0.05 was considered statistically significant.

## Results

### Baseline Characteristics

Table 1 summarizes the baseline characteristics of 297 patients with CKD. Overall, the mean age was 68.8 ± 12.9 years, and 169 (56.9%) were male. The distribution of CKD stages was 8.4% stage 3b, 59.6% stage 4, and 32.0% stage 5. Among them, 52.2% had DM, 36.7% chronic GN, 83.5% hypertension, 29.6% CV disease. The prevalence of sarcopenia was 20.2%. Patients with sarcopenia were older (*P* < 0.001); had lower BMI (*P* < 0.001), waist circumference (*P* < 0.001), hip circumference (*P* = 0.001), MAMC (*P* = 0.001), SMM (*P* < 0.001), HGS (*P* < 0.001), gait speed (*P* < 0.001), BUN (*P* < 0.001), creatinine (*P* < 0.001), UPCR (*P* = 0.001); and had higher eGFRcre (*P* < 0.001). Notably, those with sarcopenia had significantly lower SI (*P* < 0.001) and 24-h Ucr (*P* < 0.001).

**Table 1:** Demographic and clinical characteristics of the study population.

**Characteristics**	**Total (*n* = 297)**	**Sarcopenia (*n* = 60)**	**Non-sarcopenia (*n* = 237)**	***P-*value**
Age (years)	68.8 ± 12.9	77.0 ± 10.3	66.7 ± 12.6	<0.001^*^
Gender (male), *n* (%)	169 (56.9)	38 (63.3)	131 (55.3)	0.260
**CKD stages**, ***n*** **(%)**				
Stage 3b	25 (8.4)	9 (15.0)	16 (6.8)	0.121
Stage 4	177 (59.6)	33 (55.0)	144 (60.8)	
Stage 5	95 (32.0)	18 (30.0)	77 (32.5)	
**Diseases**, ***n*** **(%)**				
DM	155 (52.2)	25 (41.7)	130 (54.9)	0.068
Chronic GN	109 (36.7)	23 (38.3)	86 (36.3)	0.769
Hypertension	248 (83.5)	48 (80.0)	200 (84.4)	0.413
CV disease	88 (29.6)	21 (35.0)	67 (28.3)	0.308
Systolic BP (mmHg)	147 (131–163)	146 (132–166)	147 (131–162)	0.559
Diastolic BP (mmHg)	79 (70–86)	77 (68–85)	80 (70–87)	0.069
**Anthropometry measures**				
BMI (kg/m^2^)	26.3 ± 4.4	23.5 ± 3.6	27.0 ± 4.3	<0.001^*^
Waist circumference (cm)	92 ± 12	87 ± 10	93 ± 12	<0.001^*^
Hip circumference (cm)	96 (92–102)	94 (89–97)	97 (92–103)	0.001^*^
MAMC (cm)	23 ± 3	21 ± 2	23 ± 4	0.001^*^
Fat tissue mass (kg)	19.7 (14.9–25.2)	19.9 (15.9–25.1)	19.7 (14.8–25.2)	0.799
**Skeletal muscle measures**				
SMM (kg)	23.2 (17.5–28.5)	19.5 (14.6–22.2)	24.6 (18.3–29.3)	<0.001^*^
HGS (kg)	23.8 ± 8.9	18.8 ± 6.7	25.1 ± 9.0	<0.001^*^
Gait speed (m/s)^a^	0.93 (0.73–1.11)	0.77 (0.56–0.98)	0.96 (0.79–1.14)	<0.001^*^
**Laboratory data**
Hemoglobin (g/dL)	10.8 ± 1.8	11.0 ± 1.8	10.8 ± 1.9	0.298
Albumin (g/dL)	4.1 (3.8–4.3)	4.1 (3.9–4.3)	4.1 (3.8–4.3)	0.199
TCH (mg/dL)	146 (124–172)	143 (121–178)	146 (125–172)	0.692
Glucose (mg/dL)	106 (93–138)	107 (94–138)	106 (93–138)	0.866
BUN (mg/dL)	44 (32–58)	34 (25–55)	45 (35–59)	<0.001^*^
Creatinine (mg/dL)	2.8 (2.2–3.8)	2.3 (1.9–3.3)	2.9 (2.4–3.8)	<0.001^*^
Cystatin C (mg/L)	2.9 (2.4–3.6)	2.7 (2.2–3.5)	2.9 (2.4–3.6)	0.135
eGFRcre (mL/min/1.73 m^2^)	20 (14–26)	24 (17–32)	19 (14–25)	<0.001^*^
eGFRcys (mL/min/1.73 m^2^)	18 (13–24)	19 (13–25)	18 (13–24)	0.401
UPCR (g/g)	0.9 (0.3–2.1)	0.4 (0.2–1.3)	1.1 (0.4–2.4)	0.001^*^
SI	50.8 (42.5–61.8)	45.6 (37.0–52.1)	52.4 (44.8–63.1)	<0.001^*^
24-h Ucr (mg/day)^b^	921 (708–1,224)	734 (589–1,001)	974 (738–1,246)	<0.001^*^

*Values for continuous variables are given as means ± standard deviations or medians and interquartile ranges. Categorical variables are expressed as numbers (%). CKD, chronic kidney disease; DM, diabetic mellitus; GN, glomerulonephritis; CV, cardiovascular; BP, blood pressure; BMI, body mass index; MAMC, mid-arm muscular circumference; SMM, skeletal muscle mass; HGS, handgrip strength; TCH, total cholesterol; BUN, blood urea nitrogen; eGFRcre, estimated glomerular filtration rate from serum creatinine; eGFRcys, estimated glomerular filtration rate from serum cystatin C; UPCR, urine protein/creatinine ratio; SI, sarcopenia index; Ucr, urine creatinine excretion*.

a *Gait speed test was available in 275 patients*.

b *24-h urine sample was available in 265 patients*.

* *P < 0.05 was considered statistically significant between sarcopenia and non-sarcopenia groups*.

### Association of SI With Skeletal Muscle Measures

**Figure 1:**
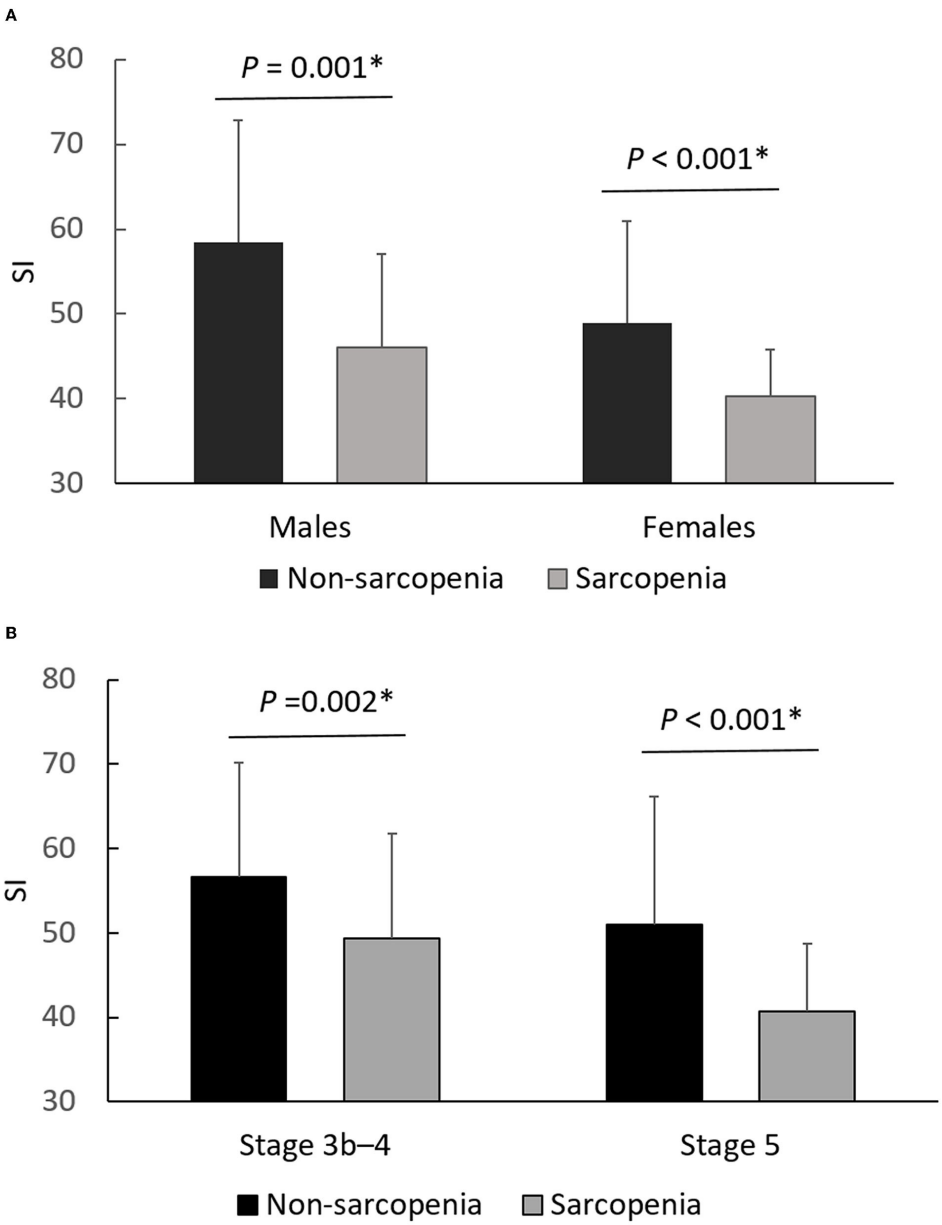
Differences of SI between non-sarcopenia and sarcopenia, stratified by gender **(A)** and CKD stage **(B)**. *P < 0.05 was considered statistically significant.

As shown in Figure 1, SI was significantly lower in the sarcopenia group than in the non-sarcopenia group in both genders (46.0 ± 11.1 vs. 58.4 ± 14.4, *P* = 0.001 in males; 40.3 ± 5.5 vs. 48.9 ± 12.0, *P* < 0.001 in females) and in different CKD stages (49.4 ± 12.4 vs. 56.6 ± 13.6, *P* = 0.002 in stage 3b-4; 40.7 ± 8.0 vs. 51.0 ± 15.1, *P* < 0.001 in stage 5).

**Figure 2:**
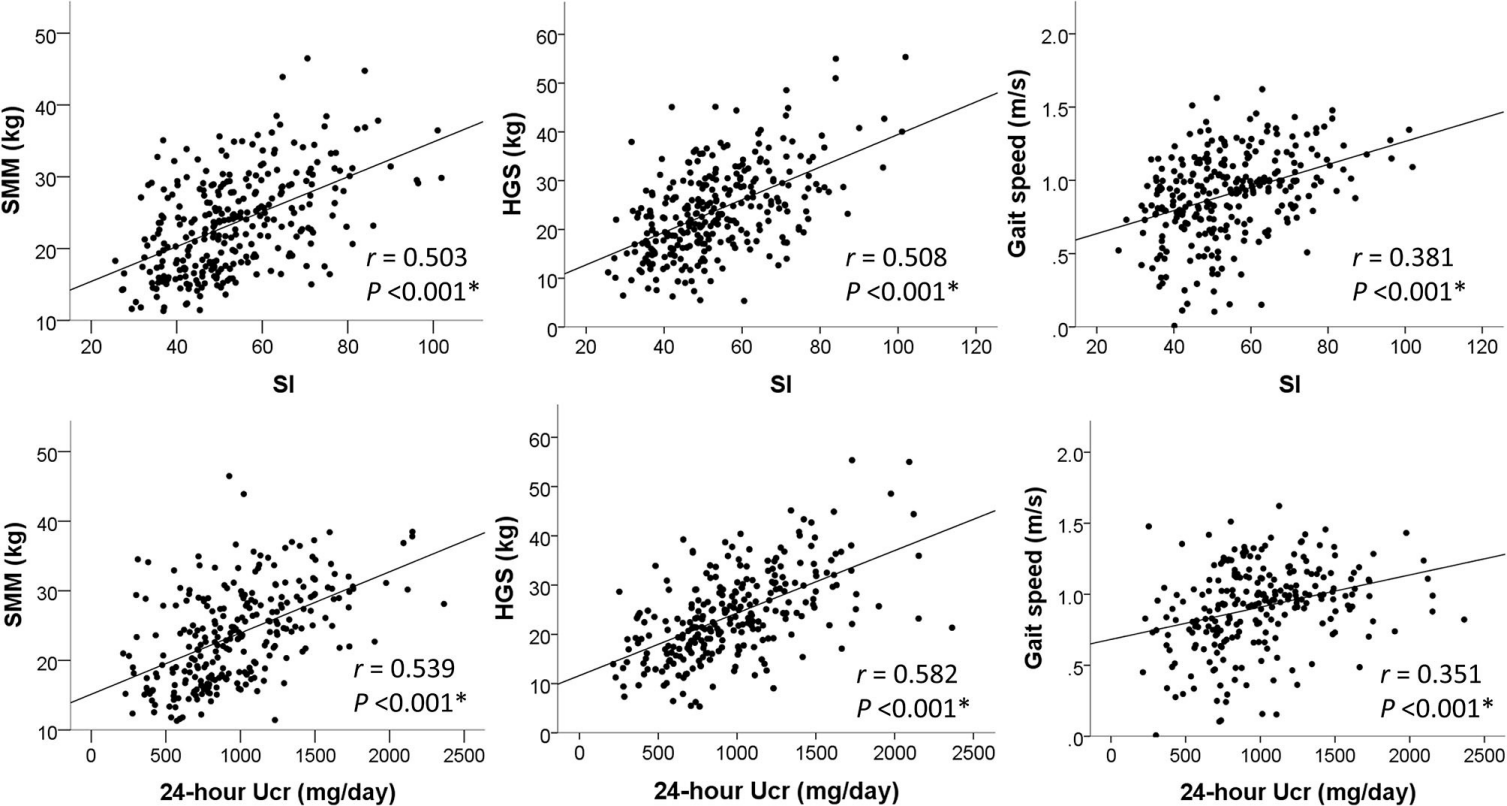
Correlations of SI and 24-h Ucr with skeletal muscle measures. *P < 0.05 was considered statistically significant.

In Figure 2, SI was positively correlated with SMM (*r* = 0.503, *P* < 0.001), HGS (*r* = 0.508, *P* < 0.001), and gait speed (*r* = 0.381, *P* < 0.001). These moderate-intensity correlations were close to those of 24-h Ucr, which yielded correlation coefficients of 0.539, 0.582, and 0.351 with SMM, HGS, and gait speed, respectively. The correlations of other proposed SI with skeletal muscle metrics are also reported in Table 2.

**Table 2:** Spearman's correlations between different proposed sarcopenia indices based on creatinine and cystatin C with skeletal muscle mass, strength, and gait speed.

**Sarcopenia indices**	**SMM (kg)**		**HGS (kg)**		**Gait speed (m/s)**	
	***r***	***P-*value**	***r***	***P-*value**	***r***	***P-*value**
Cr×eGFRcys	0.503	<0.001^*^	0.508	<0.001^*^	0.381	<0.001^*^
Cr/CysC	0.440	<0.001^*^	0.368	<0.001^*^	0.313	<0.001^*^
Serum Cr	0.362	<0.001^*^	0.101	0.083	0.179	0.003^*^
eGFRcys-eGFRcre	0.142	0.015^*^	0.169	0.003^*^	0.251	<0.001^*^
eGFRcys/eGFRcre	0.124	0.033^*^	0.167	0.004^*^	0.240	<0.001^*^

*SMM, skeletal muscle mass; HGS, handgrip strength; Cr, creatinine; eGFRcys, estimated glomerular filtration rate from serum cystatin C; CysC, cystatin C; eGFRcre, estimated glomerular filtration rate from serum creatinine*.

* *P < 0.05 was considered statistically significant*.

Simple and multiple linear regression analyses of the SI in relation to SMM, HGS, and gait speed are shown in Table 3. In the unadjusted model (model 1), SI was significantly associated with SMM, HGS, and gait speed, the results of which were sustained after extensive adjustment for potential confounders, including age, sex, DM, hypertension, CV disease, BMI, waist and hip circumference, hemoglobin, albumin, TCH, glucose, eGFRcys, and UPCR (model 4).

**Table 3:** Independency of SI with SMM, HGS, and gait speed.

**Variables**	**SI (per 1-SD increase)**	
	**β (95% CI)**	***P-*value**
**SMM (kg)**
Model 1	3.44 (2.78–4.11)	<0.001^*^
Model 2	1.08 (0.53–1.62)	<0.001^*^
Model 3	1.11 (0.62–1.60)	<0.001^*^
Model 4	1.52 (1.02–2.01)	<0.001^*^
**HGS (kg)**
Model 1	4.74 (3.87–5.60)	<0.001^*^
Model 2	2.78 (1.89–3.66)	<0.001^*^
Model 3	2.78 (1.89–3.67)	<0.001^*^
Model 4	2.77 (1.81–3.74)	<0.001^*^
**Gait speed (m/s)**
Model 1	0.11 (0.08–0.14)	<0.001^*^
Model 2	0.06 (0.02–0.09)	0.002^*^
Model 3	0.06 (0.02–0.09)	0.002^*^
Model 4	0.05 (0.01–0.09)	0.014^*^

*Model 1, Unadjusted analysis. Model 2, Adjusted for age and sex. Model 3, Model 2 additionally adjusted for diabetes mellitus, hypertension, cardiovascular disease, body mass index, waist and hip circumference. Model 4, Model 3 additionally adjusted for hemoglobin, albumin, total cholesterol, fasting glucose, estimated glomerular filtration rate based on cystatin C, and urine protein to creatinine ratio. SI, sarcopenia index; SMM, skeletal muscle mass; HGS, handgrip strength*.

* *P < 0.05 was considered statistically significant*.

### Diagnostic Performance of SI on Sarcopenia

The AUC, cut-off values, sensitivity, specificity, PPV, and NPV of SI for sarcopenia are shown in Table 4. The SI had acceptable discriminative power in both males [AUC 0.646, 95% confidence interval (CI) 0.569–0.718, *P* = 0.003] and females (AUC 0.754, 95% CI 0.670–0.826, *P* < 0.001). In males, the best cut-off was 53.9, which provided 71.1% sensitivity, 58.0% specificity, 32.9% PPV, and 87.4% NPV; in females, the best cut-off was 45.8, which provided 81.8% sensitivity, 62.3% specificity, 31.0% PPV, and 94.3% NPV. The diagnostic performance of 24-h Ucr was also provided.

**Table 4:** Diagnostic validity of SI and 24-h Ucr on sarcopenia, overall and stratified by gender.

**Low SI**						
	**AUC (95% CI)**	**Cut-off**	**Sen (%)**	**Spe (%)**	**PPV (%)**	**NPV (%)**
Overall	0.659 (0.602–0.713)^*^					
Male	0.646 (0.569–0.718)^*^	53.9	71.1	58.0	32.9	87.4
Female	0.754 (0.670–0.826)^*^	45.8	81.8	62.3	31.0	94.3
**Low 24-h Ucr^a^**						
Overall	0.659 (0.599–0.716)^*^					
Male	0.688 (0.608–0.761)^*^	1022	72.4	61.8	30.9	90.5
Female	0.692 (0.599–0.776)^*^	710	80.0	64.5	32.7	93.7

*SI, sarcopenia index; Ucr, urine creatinine excretion; AUC, area under the ROC curves; CI, confidence interval; Sen, sensitivity; Spe, specificity; PPV, positive predictive value; NPV, negative predictive value*.

a *24-h urine sample was available in 265 patients*.

* *P < 0.05 was considered statistically significant*.

## Discussion

In our study, the novel SI, Cr×eGFRcys, was independently associated with skeletal muscle mass, strength, and usual gait speed in non-dialysis advanced CKD. The correlation coefficients with muscle measures and the discriminatory power for sarcopenia exhibited by SI were similar to the performance of 24-h Ucr.

Sarcopenia is concerning in patients with advanced-stage CKD; thus, assessing skeletal muscle health is of the same importance as monitoring renal function change. Considering the limited feasibility of imaging studies, several novel biomarkers, such as myokines, inflammatory and oxidative markers, have been emergently reported for skeletal muscle estimation (26, 27). Unfortunately, there is a gap between these results and their translation into real-world practice, given their weak correlations with skeletal muscle mass and high measurement cost. To our knowledge, 24-h Ucr, which was first validated in 1983, remains the most reliable and robust marker for the prediction of skeletal muscle mass (11). In patients with CKD, low 24-h Ucr is associated with reduced skeletal muscle mass, frailty, and enhanced mortality risks (28–30). Our study showed moderate correlations of 24-h Ucr with skeletal muscle mass and strength in advanced CKD, which were in line with a previous report from the large-scale Chronic Renal Insufficiency Cohort (CRIC), showing a correlation coefficient of 0.5 between 24-h Ucr and skeletal muscle mass, as evaluated by either BIA or DEXA in patients with CKD. This correlation was stronger in those with proper urine collection (30). Nevertheless, poor collection of urine samples is common in outpatient settings. As indicated in the CRIC cohort, up to one-third of urine samples were regarded as poor quality, which justifies the development of alternative surrogate markers to monitor skeletal muscle health in patients with advanced-stage CKD.

For the first time, we demonstrated that the novel SI calculated from creatinine and cystatin C, two widely used renal markers, independently predicts skeletal muscle mass, muscle strength, and physical performance in advanced-stage CKD, even after adjusting for potential confounders extensively. Cut-off values for the novel SI yielded high NPV, which suggested its potential use for screening sarcopenia in patients with advanced-stage CKD. In addition, our previous work showed a close relationship between low SI and all-cause mortality in real-world cases using our CKD database (18). The patients in the low SI group conferred a three-fold increased mortality hazard after full adjustment for risk factors in comparison with those in the normal SI group.

Given the decreased serum creatinine, but not cystatin C, in patients experiencing muscle wasting, other indices based on these two renal markers, such as the creatinine-to-cystatin C ratio (Cr/CysC), eGFRcys-to-eGFRcre ratio (eGFRcys/eGFRcre), and the difference between eGFRcys and eGFRcre (eGFRcys–eGFRcre), have been reported for the assessment of sarcopenia in various populations (31–39). Our study demonstrated that, among these serum indices, Cr×eGFRcys exhibited the best correlations with skeletal muscle mass, strength, and gait speed. This observation was in accordance with results from two recent studies in cancer patients, which showed that Cr×eGFRcys outperformed Cr/CysC in predicting sarcopenia and postoperative complications (40, 41).

Our study is the first to explore the clinical utility of Cr×eGFRcys as an SI for predicting sarcopenia in advanced-stage CKD patients. The strength of the study was that 24-h Ucr was collected simultaneously, ensuring a direct comparison between the novel SI and 24-h Ucr. However, we report significant limitations. First, the sample size was relative limited, which precluded our further stratification by each CKD stage. The application of the gender-specific SI cut-offs in each CKD stage was shown in Supplementary Table 1. Second, the discriminatory power of SI in men was low, which limited its clinical utility to predict sarcopenia. Third, a single-frequency BIA was used to measure skeletal muscle mass, which could be overestimated by hydration status in patients with advanced-stage CKD. However, a strong correlation and good agreement between the BIA with dual-energy X-ray absorptiometry was demonstrated in dialysis patients (42). Fourth, non-renal factors other than skeletal muscle mass, including dietary protein intake, physical activity, inflammation, obesity, endocrine disease, and certain medications, affect creatinine or cystatin metabolism (43, 44) and account for variations. Thus, whether Cr×eGFRcys is useful for dynamic monitoring of skeletal muscle change longitudinally and for evaluation of response to intervention should be determined in future studies. Fifth, the quality of urine collection could not be ascertained. We propose that 24-h Ucr is the most reliable clinical marker for sarcopenia when an accurate collection of urine samples is ensured. Sixth, although the criteria for sarcopenia were well-developed in the geriatric population, there was a lack of agreement among patients with CKD. Finally, this is a single-center study in Taiwan; therefore, our findings should be extrapolated with caution, especially to other ethnic populations.

## Conclusion

In conclusion, our study demonstrated that Cr×eGFRcys was independently associated with skeletal muscle mass, strength, and usual gait speed in non-dialysis advanced CKD. In addition to providing more accurate renal function estimates, measuring serum creatinine and cystatin simultaneously to generate the novel SI, Cr×eGFRcys, may be an easy-to-use approach for screening skeletal muscle health in patients with advanced-stage CKD. However, large-scale studies are encouraged to extend our findings.

## Data Availability Statement

The raw data supporting the conclusions of this article will be made available by the authors, without undue reservation.

## Ethics Statement

The studies involving human participants were reviewed and approved by Hualien Tzu Chi Hospital (108-219-A). The patients/participants provided their written informed consent to participate in this study.

## Author Contributions

Y-LL: conceptualization, methodology, formal analysis, and writing—original draft preparation. C-HW and I-CC: investigation. I-CC: data curation. B-GH: writing—review and editing and supervision. All authors reviewed the manuscript, contributed to the article, and approved the submitted version.

## Funding

Grants from the Hualien Tzu Chi Hospital and Buddhist Tzu Chi Medical Foundation, Hualien, Taiwan (TCRD 110-47) supported this study.

## Conflict of Interest

The authors declare that the research was conducted in the absence of any commercial or financial relationships that could be construed as a potential conflict of interest.

## Publisher's Note

All claims expressed in this article are solely those of the authors and do not necessarily represent those of their affiliated organizations, or those of the publisher, the editors and the reviewers. Any product that may be evaluated in this article, or claim that may be made by its manufacturer, is not guaranteed or endorsed by the publisher.
