# Supplementary material for: A Novel Application of Serum Creatinine and Cystatin C to Predict Sarcopenia in Advanced CKD
Source: Front Nutr. 2022 Feb 25;9:828880. doi: 10.3389/fnut.2022.828880 (PMC8914226; doi:10.3389/fnut.2022.828880)
Supplement: Supplementary file 1 [file Table_1.DOCX]

Supplementary Material

**Supplementary Table 1.** The diagnostic performance of SI on sarcopenia in different CKD stages, using gender-specific SI cut-offs developed in the study.

| Stage 3b-4 | | Sarcopenia | | |  | Sensitivity = 64.3%  Specificity = 66.3%  PPV = 33.3%  NPV = 87.6% |
| --- | --- | --- | --- | --- | --- | --- |
|  |  | Present |  | Absent | Total |  |
| SI | Low | 27 |  | 54 | 81 |  |
|  | Normal | 15 |  | 106 | 121 |  |
|  | Total | 42 |  | 160 | 202 |  |
| Stage 5 | | Sarcopenia | | |  | Sensitivity = 88.9%  Specificity = 46.8%  PPV = 28.1%  NPV = 94.7% |
|  |  | Present |  | Absent | Total |  |
| SI | Low | 16 |  | 41 | 57 |  |
|  | Normal | 2 |  | 36 | 38 |  |
|  | Total | 18 |  | 77 | 95 |  |
